# Joint association of polysocial risk score and lifestyle with incident essential hypertension: a prospective cohort study in the UK biobank

**DOI:** 10.1186/s12872-025-04930-2

**Published:** 2025-07-04

**Authors:** Yumei Zhao, Yingbai Wang, Zihan Xu, Jiaofeng Xiang, Chuxun Zhou, Xiaolin Li, Shicai Ye, Suru Yue, Xuefei Hou, Jia Wang, Jiayuan Wu

**Affiliations:** 1 Clinical Research Service Center, Affiliated Hospital of Guangdong Medical University, Zhanjiang, Guangdong, 524001 China; 2 Collaborative Innovation Engineering Technology Research Center of Clinical Medical Big Data Cloud Service in Medical Consortium of West Guangdong Province, Affiliated Hospital of Guangdong Medical University, Zhanjiang, 524001 Guangdong China; 3 Department of Gastroenterology, Affiliated Hospital of Guangdong Medical University, Zhanjiang, Guangdong, 524001 China; 4 Affiliated Hospital of Guangdong Medical University, No. 57 South of Renmin Avenue, Zhanjiang, 524001 China

**Keywords:** Essential hypertension, Polysocial risk score, Lifestyle, Interaction, UK biobank

## Abstract

**Background:**

The polysocial risk score (PsRS) estimates cumulative social vulnerability. While social factors and lifestyles are linked to essential hypertension (EH), their combined effects are unclear. This study aims to explore the independent and joint associations of social vulnerability and lifestyle with EH in the UK Biobank Study.

**Methods:**

The study included 131,154 UK Biobank participants without EH at baseline. PsRS was calculated from 14 social determinants across three risk categories: socio-economic, psychological, and environmental factors, all significantly linked to EH development after Bonferroni adjustment. The healthy lifestyle score was based on smoking, alcohol, physical activity, diet, and sleep. The Cox proportional hazards model with HR and 95% CI analyzed PsRS and lifestyle effects on EH incidence, and interactions between PsRS and lifestyle score were assessed additively and multiplicatively.

**Results:**

Over an average follow-up of 13.5 years, 19,281 participants (14.7%) developed EH. After adjusting for confounders, participants with intermediate (5,–7) and high (≥ 8) PsRS exhibited increased EH risks with HRs of 1.06 (95% CI: 1.02–1.10) and 1.15 (95% CI: 1.11–1.20), respectively, compared to those with low PsRS (≤ 4). In the fully adjusted model, every one-point increase in PsRS was associated with a 7.0% higher risk of EH (HR = 1.07, 95% CI: 5.0–9.0, *P* for trend < 0.001). Intermediate (2–3) and favorable (4–5) lifestyle scores were associated with lower EH risks, with HRs of 0.86 (95% CI: 0.82–0.90) and 0.77 (95% CI: 0.73–0.81), respectively, compared to an unfavorable lifestyle score (0–1). Each one-point decrement in lifestyle score was corresponded to a 14% reduced in EH risk (HR = 0.86, 95% CI: 0.84–0.89, *P* for trend < 0.001). Joint effects analysis revealed significant synergistic interactions with individuals possessing both high PsRS and unfavorable lifestyles experienced the greatest risk (HR = 1.47,95% CI: 1.37–1.58). Additive interaction metrics confirmed this synergy, indicating 66% of EH risk in this subgroup stemmed from PsRS-lifestyle interaction. Multiplicative interactions were likewise significant (*P*_interaction_<0.001).

**Conclusion:**

An unhealthy lifestyle may exacerbate the impact of social vulnerability on EH risk. Modifying both social vulnerability and lifestyle factors could reduce EH incidence.

**Supplementary Information:**

The online version contains supplementary material available at 10.1186/s12872-025-04930-2.

## Introduction

Essential hypertension (EH), also known as idiopathic hypertension, is a common preventable risk factor for cardiovascular disease and premature death [1]. The global morbidity of EH has steadily increased worldwide in recent years [2]. An analysis of EH prevalence trends shows that the number of EH cases aged 30 to 79 years has doubled worldwide from 1990 to 2019, with the number of women increasing from 331 million in 1990 to 626 million in 2019 and the number of men increasing from 317 million in 1990 to 652 million in 2019 [3]. Global epidemiological studies on hypertension have revealed that hypertension-related deaths have reached 8.5 million, with 88% of these fatalities occurring in low- and middle-income countries [2]. According to the World Health Organization, only 54% of hypertensive patients are diagnosed, 42% receive treatment, and merely 21% achieve hypertension control [4]. EH is therefore not only a widespread disease but also a major public health burden worldwide.

Social determinants of health (SDOH) are the conditions in the environments in which people are born, live, learn, work, play, worship, and age that influence a wide range of health, functioning, and quality of life, as well as the risks of developing diseases and premature death. Healthy People 2030 (https://health.gov/healthypeople), a national health promotion initiative spearheaded by the U.S. Department of Health and Human Services (HHS), aims to enhance public health and well-being through data-driven objectives and reduce health disparities. Healthy Population 2030 outlines five key areas of SDOH, namely economic stability, access to and quality of education, access to and quality of healthcare, neighborhood and built environment, and social and community context. In addition to genetic susceptibility, several SDOHs, such as economic stability and environmental conditions, have been identified as risk factors for EH [5]. To comprehensively assess these systemic factors, we introduced a Polysocial Risk Score (PsRS), a novel metric based on the 2030 Healthy Population Initiative guidelines [6]. The PsRS integrates 17 pre-selected SDOH categorized into three domains: socio-economic, social psychological, and social environmental [6]. Disadvantaged social status is a very important upstream determinant of health and even more important than access to health services [7]. Previous research has recognized the significant influence of certain social determinants, including wealth and income, education and employment, or occupational status, on the morbidity of EH [8].

Unhealthy lifestyles are significantly associated with disproportionate harm in disadvantaged populations, and a significant association between lifestyle and disadvantage for cardiovascular disease mortality has been observed [9]. Moreover, EH is strongly influenced by behavioral lifestyles, which mainly include overweight/obesity, unhealthy diet, excessive sodium, and insufficient potassium intake, insufficient physical activity, smoking, and alcohol consumption [10]. Epidemiological studies have shown that the risk of EH is positively correlated with body mass index (BMI) and gradually increases from underweight to obesity [11]. With globalization, the Western diet has become a trend in many developing countries. The Western diet is usually high in sodium and can impair vascular endothelial function and alter vascular elasticity, which is also an important risk factor for EH [12]. In addition, people who do not exercise regularly have a higher risk of EH than those who do [13]. Thus, different lifestyles have different effects on EH risk, and combinations of lifestyle factors may increase the association with chronic disease and mortality compared to a single lifestyle factor.

The etiology of EH is undoubtedly multifactorial. The combined effects of risk factors on EH need to be identified to ensure that interventions are targeted at high-risk populations. To date, most of the available data have examined the separate effects of social or lifestyle factors on EH risk [9, 13, 14]. Combinations of risk factors in particular have a synergistic effect and increase morbidity and mortality [15]. However, the effects of combinations of social factors on EH risk have not been investigated, and the joint effect of social factors and lifestyle on EH risk remains uncertain. Therefore, this study aimed to investigate the synergistic effect between multiple social risks and a healthy lifestyle on EH risk using the PsRS and the Healthy Lifestyle Score.

## Methods

### Study participants

The detailed research design and characteristics of the UK Biobank (www.ukbiobank.ac.uk) have been elaborated in previous studies [16]. This study is a population-based prospective cohort study, which involves more than half a million participants from the general population in Scotland, England, and Wales between March 2006 and October 2010. The recruitment was carried out via postal invitation. Participants aged 40–69 who are registered with the National Health Service (NHS) and live within 25 miles of one of 22 research assessment centers were invited to join the UK Biological Cohort Study. After providing written informed consent, the participants were asked to complete nurse-administered touch-screen questionnaires on diet, lifestyle, and healthy relevant information, undergo comprehensive physical examinations, and provide biological samples.

The present study was carried out under the UK Biobank Data Resource Application number 97,101. The flow chart of UK Biobank participants for this study is shown in Supplementary Fig. 1. We extracted data from 502,379 participants, after excluding persons diagnosed with EH disease at baseline and those who lacked data for PsRS or healthy lifestyle scores calculation. In total, 131,154 participants were eligible for analysis. In this study, all exposure factors and covariates were collected at the initial baseline assessment (touch-screen questionnaire, online questionnaire, or verbal interview).

### Diagnosis of EH

The follow-up period extended from the initial enrollment phase (March 2006 to October 2010) in Scotland, England, and Wales until either the occurrence of EH onset or the pre-specified data lock point (February 1, 2023) through linked national health registries, whichever occurred earlier. A new diagnosis of EH was obtained from the admission registries using the International Classification of Diseases 10 diagnosis code I10–I13. At baseline, the participants of the UK Biobank study had their blood pressure measured in a quiet state using the Omron HEM-7015IT digital blood pressure monitor, a second measurement was performed after 1 min of rest, and the average of the two measurements was taken as the baseline blood pressure value. According to the guidelines of the European Society of Cardiology/European Society of Hypertension [17], EH was defined as systolic blood pressure (SBP) ≥ 140 mmHg and/or diastolic blood pressure ≥ 90 mmHg.

### Calculation of PsRS

Based on the 2030 Healthy Population Initiative guidelines and previous studies, 17 social determinants were pre-selected in this study, which were categorized into three distinct groups: socio-economic, social psychological, and social environmental factors [6]. Details of these determinants are provided in Supplementary Table 1. If the social determinants were significantly related to EH event with a Bonferroni’s corrected *P* values < 0.003 (0.05/17 comparisons) in a fully adjusted Cox regression analysis with adjustment for age, sex, race, body mass index (BMI), smoking status, alcohol intake, physical activity, diet quality score, and sleep quality score, they will be incorporated into the calculation of PsRS. Higher PsRS values indicate greater social vulnerability. Further details can be found in the Supplementary Information.

### Assessment of healthy lifestyle score

Based on previous literature, a healthy lifestyle score was set according to five behavioral lifestyle factors, namely, smoking status, alcohol consumption, sports activities, food, and sleep quality [18]. Weekly alcohol intake was assessed via a touch-screen questionnaire. Physical activity was evaluated based on the frequency and duration of various exercises. Diet was evaluated by calculating the diet quality score ranged from 0 to 7 based on their intake of greens, fruits, whole grains, fish, processed meat, refined grains, and mammalian meat. Sleep quality was scored from 0 to 5 based on factors like sleep duration and insomnia frequency. For healthy lifestyle scores, participants were given one points if they met one of the following criteria: [1] never smoked; [2] moderate or never alcohol use (< 3 times/week); [3] sufficient physical activity (satisfy any one criteria: modest activity ≥ 5 times/week or ≥ 150 min/week, or vigorous activity ≥ 1 time/week or ≥ 75 min/week, or ≥ 150 min/week of combined moderate and vigorous activity); [4] a diet score of ≥ 4, and [5] a sleep score of ≥ 4. The overall lifestyle scores ranged from 0 to 5, categorizing lifestyles as unfavorable (0 − 1), intermediate (2 − 3), or favorable (4 − 5). Further details can be found in the Supplementary Information.

### Covariates

Age, sex, race, BMI, waist circumference, lipid-lowering drugs use, hypoglycemic medications use, diabetes, and adiposity were adjusted as potential confounders in the Cox proportional hazards model. BMI was calculated based on the measured height and weight during the initial assessment center visit. Adiposity was defined as BMI ≥ 30 kg/m^2^ or males with waist circumference > 85 cm or females with waist > 80 cm [19, 20].

### Statistical analysis

Continuous data are expressed in terms of mean and standard deviations (SDs), or median and quartile intervals (IQRs), and disaggregated data are expressed as case numbers with percentages. One-way ANOVA test or *χ*^2^ test was used for between-group comparison of baseline data where appropriate. The Cox proportional risk model with hazard ratio (HR) and 95% confidence interval (CI) was used to analyze the relationship between PsRS or healthy lifestyle score and the risk of EH incidence. The time between the baseline survey and occurrence time was taken as the time scale. The proportional hazard assumption was examined by plotting Schoenfeld residuals, and no evidence of serious violation was observed. The unadjusted model only included PsRS or lifestyle scores without adjustment of any covariate. The fully adjusted model was adjusted for sex, age, race, BMI, waist circumference, alcohol consumption, smoking status, physical activity, diet pattern, diet quality score, sleep quality lipid-lowering drugs use, hypoglycemic medications use, diabetes, and adiposity for PsRS, and was adjusted for age, sex, race, BMI, waist circumference, lipid-lowering drugs use, hypoglycemic medications use, diabetes, and adiposity for healthy lifestyle score. The adjusted variables mentioned above do not exhibit multicollinearity. A value of *P* for the trend was computed, in which PsRS or healthy lifestyle score was taken as a continuous variable. The potential nonlinear relationship between PsRS or healthy lifestyle score and EH risk was tested by restricted cubic splines for multivariate Cox regression models, and with knots placed at the 10th, 50th, and 90th percentiles of PsRS or healthy lifestyle score. The Kaplan–Meier method was used for calculating the 5-year cumulative morbidity of EH per 1,000 person-years.

The additive and multiplicative interaction effect of between PsRS and lifestyle scores for EH events was also analyzed. For additive interaction, a new term with 9 categories (3 × 3) was formulated based on the 3 levels of PsRS and the 3 categories of lifestyle scores by using the participants with the low level of PsRS and the favorable level of lifestyle score as the reference group. Moreover, relative excess risks due to interaction (RERIs), attributable proportion due to interaction (AP), and synergy index (SI) were applied to quantify the interaction on the additive scale. For the multiplicative interaction, we created a product term between PsRS and lifestyle score. Likelihood tests were applied to assess the significance of the interaction term by comparing the model with and without the interaction term. A *P* value less than 0.05 of the interaction term suggests multiplicative interaction.

We employed a causal mediation analysis approach to investigate the potential mediating role of lifestyle scores in the association between PsRS and EH. Within a counterfactual causal mediation analysis framework based on Cox regression, we systematically evaluated the total effect, direct effect, and indirect effect of PsRS on EH, with the stability of results enhanced by 1000 bootstrap resamples. The proportion of mediated effect was subsequently calculated.

Four additional analyses were performed as sensitivity analyses to test the stability of the results. The tests include the exclusion of EH cases occurring during the first 5 years of follow-up, the exclusion of participants who were obese (BMI ≥ 28 kg/m^2^) at baseline, the exclusion of prehypertensive individuals at baseline, and the calculation of physical inactivity and present/past smoking as social determinants into the PsRS based on the Healthy People 2030 Initiative [6]. Statistical analysis was performed using R software. Statistical significance was set at *P* < 0.05.

## Results

### Ascertainment of PsRS and healthy lifestyle score

According to the fully adjusted model, 14 of the 17 social determinants were significantly correlated with the risk of EH (Supplementary Table 2). Multicollinearity among these components was generally low (all VIFs < 5; pairwise|r| <0.5), except for a strong correlation between Greenspace remote and Natural environment remote (*r* = 0.865), which reflected their conceptual overlap as environmental measures (Supplementary Tables 4–5). Accordingly, these 14 factors were included in the calculation of PsRS, and the values ranged from 0 to 14. The proportion of participants exposed to various SDOH ranged from 14.90 to 58.2%, but most of the values were around 50%. Among the 14 social determinants factors included in the PsRS calculations, fully adjusted HR values ranged from 0.99 (95% CI: 0.93–1.05) for blue space remote to 1.41 (95% CI: 1.26–1.56) for emotional distress.

Similarly, the proportion of participants exposed to different healthy lifestyle factors ranged from 40.90 to 96.30% with an average of 58.48%. Based on the fully adjusted Cox regression models, all five healthy lifestyles were protective factors for preventing EH (HR < 1), and the values ranged from 0.62 (95% CI: 0.59–0.65) for never-smoking to 0.91 (95% CI: 0.89–0.94) for healthy sleep (Supplementary Table 6).

### Baseline characteristics of PsRS categories

The baseline features of participants in different PsRS categories are shown in Table 1. Among the 131,154 participants, low (PsRS ≤ 4), medium (PsRS 5–7), and high (PsRS ≥ 8) PsRS groups accounted for 24.8% (*n* = 32,566), 50.9% (*n* = 66,754), and 24.3% (*n* = 31,834) of the whole population, respectively. Participants in the low-PsRS group had lower BMI, had a higher proportion of never smoked and low-to-moderate alcohol consumption, and had a better diet and sleep quality than those in the intermediate- or high-social-risk groups (all *P* values of inter-group < 0.001).

**Table 1:** Baseline characteristics of participants

Variable	Overall	Polysocial risk score			
		Low	Intermediate	High	*P* for inter-group
Participants, n (%)	131,154	32,566 (24.8%)	66,754 (50.9%)	31,834 (24.3%)	< 0.001
Age (years)	54.54 ± 8.01	54.7 ± 7.60	54.7 ± 8.06	54.16 ± 8.29	< 0.001
Male, n (%)	32,566	15,644 (48%)	29,559 (44.3%)	12,945 (40.7%)	< 0.001
White, n (%)	119,377	30,758 (94.4%)	60,882 (91.2%)	277,377 (87.1%)	< 0.001
BMI (kg/m^2^)	26.48 ± 4.29	26.23 ± 4.0	26.43 ± 4.22	26.85 ± 4.73	< 0.001
Waist circumference (cm)	87.82 ± 12.57	87.47 ± 12.17	87.66 ± 12.50	88.48 ± 13.09	< 0.001
SBP (mmHg)	134.72 ± 18.01	135.79 ± 18.02	134.79 ± 18.02	133.48 ± 17.92	< 0.001
Never smoked, n (%)	75,212	20,574 (63.2%)	38,511 (57.7%)	16,127 (50.7%)	< 0.001
Low to moderate alcohol intake, n (%) ^a^	67,364	14,911 (45.8%)	33,776 (50.6%)	18,677 (58.7%)	< 0.001
Adequate physical activity, n (%) ^b^	126,240	31,393 (96.4%)	64,370 (96.4%)	30,476 (95.7%)	< 0.001
Diet quality score	3.43 ± 1.15	3.46 ± 1.11	3.44 ± 1.41	3.37 ± 1.20	< 0.001
Sleep quality score	3.24 ± 0.97	3.35 ± 0.95	3.25 ± 0.96	3.10 ± 0.99	< 0.001
Lipid-lowering drugs use	11,529	2620 (8%)	5845 (8.8%)	3064 (9.6%)	< 0.001
Hypoglycemic medications use	240	52 (0.2%)	103 (0.2%)	83 (0.3%)	< 0.001
Diabetes	5708	1119 (3.3%)	2749 (4.1%)	1840 (5.8%)	< 0.001
Adiposity	9169	1799 (5.5%)	4471 (6.7%)	2899 (9.1%)	< 0.001

^a^ Low to moderate alcohol intake was defined as less than three times a week

^b^ Adequate physical activity was defined as ≥ 150 min/week of moderate activity, or ≥ 75 min/week of vigorous activity, or ≥ 150 min/week of combined moderate and vigorous activity, ≥ 5 times/week of moderate activity, or ≥ 1 time/week of vigorous activity

*BMI* Body mass index, *SBP* Systolic blood pressure

###  PsRS and incident EH

**Fig. 1:**
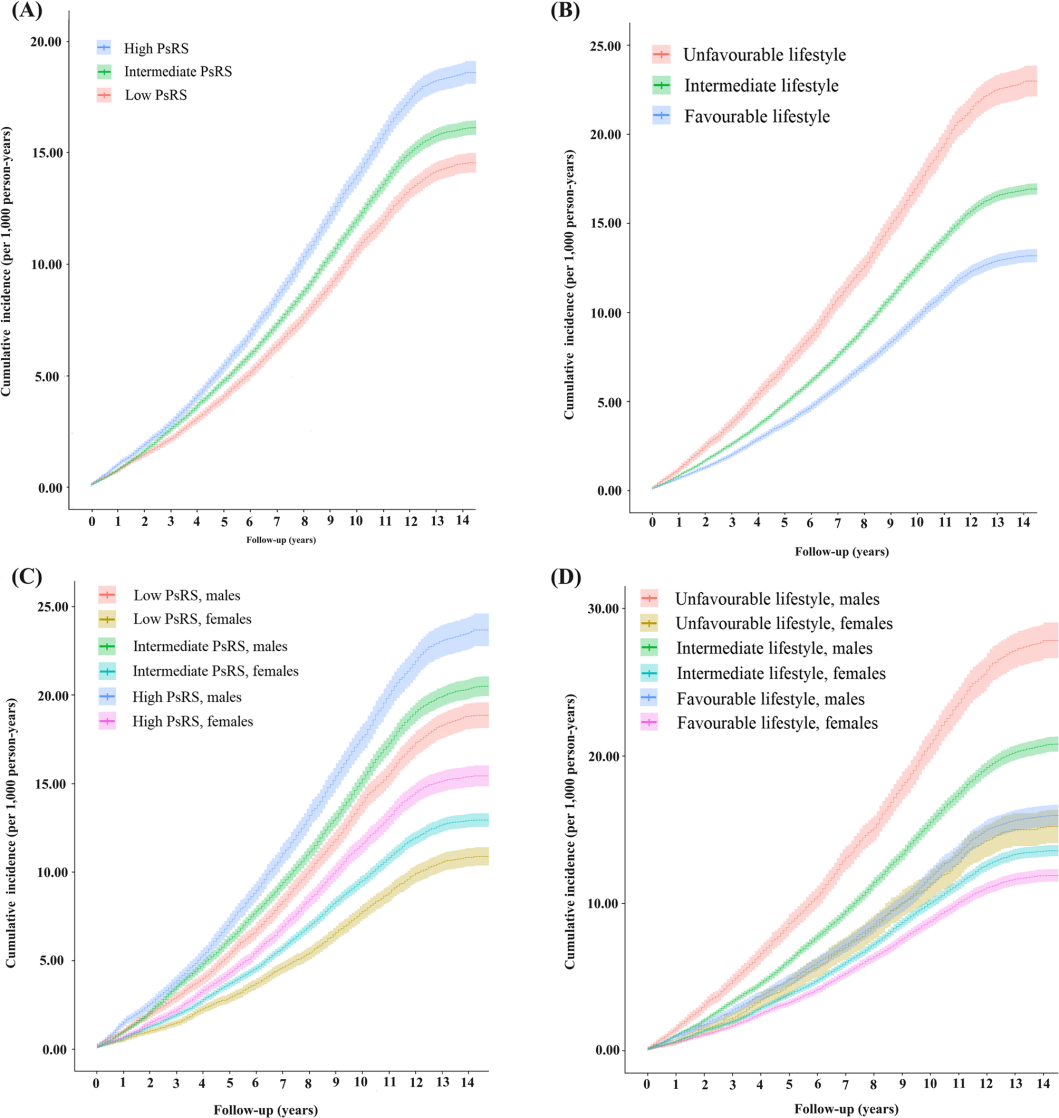
Cumulative incidence of essential hypertension stratified by PsRS group (**A**), lifestyle score group (**B**), sex-specific PsRS group (**C**), and sex-specific lifestyle score group (**D**) PsRS, polysocial risk score

The median follow-up time was 13.5 years (IQR: 12.7–14.2; 1,650,309 person-years), with 19,281 EH events among 131,154 participants. As shown in Fig. 1A, a higher PsRS is associated with a higher risk of developing EH (*P* for log-rank test < 0.001). The 5-year cumulative incidence rates of EH were 4.71 (95% CI: 4.50–4.89), 5.48 (95% CI: 5.07–5.89), and 6.41 (95% CI: 6.04–6.80) per 1,000 person-years for participants in the low-, middle-, and high-PsRS groups, respectively (Fig. 1A). After stratification based on sex, the cumulative risk of EH was consistently highest in the high-PsRS group, followed by the intermediate-PsRS group for both males and females (Fig. 1C, Supplementary Table 7). Moreover, males consistently had a higher EH risk than females in each PsRS group (Fig. 1C).

As shown in Table 2, a crude model revealed that elevated PsRS was significantly associated with an increasing risk of EH, with HRs of 1.10 (95% CI: 1.06–1.14) for the intermediate-PsRS group and 1.28 (95% CI: 1.23–1.34) for the high-PsRS group. Similarly, a fully adjusted model showed a significantly higher risk of EH in the intermediate- and high-PsRS groups compared with the low-PsRS group, with HRs of 1.06 (95% CI: 1.02–1.10) and 1.15 (95% CI: 1.11–1.20), respectively. In the fully adjusted model, the risk of EH increased by 7.0% (95% CI: 5.0–9.0%) for every point increment in PsRS (*P* for trend < 0.001). In subgroup analysis, the association of PsRS and EH risk remained stable in participants with different levels of lifestyle scores (Supplementary Fig. 2).

**Table 2:** Association of polysocial risk score and healthy lifestyle score with incident essential hypertension

Polysocial risk score	Low (≤ 4)	Intermediate (5–7)	*P* value	High (≥ 8)	*P* value	Per point increment	*P* for trend
Cases	4315 (13.3%)	9709 (14.5%)		5257 (16.1%)			
Person-years	417,782	839,436		393,091			
Unadjusted HR (95% CI)	1.00	1.10 (1.06–1.14)	< 0.001	1.28 (1.23–1.34)	< 0.001	1.13 (1.11–1.15)	< 0.001
Fully adjusted HR (95% CI) ^a^	1.00	1.06 (1.02–1.10)	< 0.001	1.15 (1.11–1.20)	< 0.001	1.07 (1.05–1.09)	< 0.001
Healthy lifestyle score	Unfavourable (0–1)	Intermediate (2–3)	*P value*	Favourable (4–5)	*P value*	Per point increment	*P* for trend
Cases	2767	11,517		4997			
Person-years	168,463	952,126		529,720			
Unadjusted HR (95% CI)	1.00	0.73 (0.70–0.76)	< 0.001	0.57 (0.54–0.60)	< 0.001	0.76 (0.74–0.78)	< 0.001
Fully adjusted HR (95% CI) ^b^	1.00	0.86 (0.82–0.90)	< 0.001	0.77 (0.73–0.81)	< 0.001	0.86 (0.82–0.89)	< 0.001

HRs and 95% CIs were calculated using a fully adjusted Cox proportional hazards model

^a^ The fully adjusted model was adjusted for age, sex, race, body mass index, waist circumference, smoking status, alcohol intake, physical activity, diet quality score, sleep quality score, lipid-lowering drugs use, hypoglycemic medications use, diabetes, and adiposity

^b^ The fully adjusted model was adjusted for age, sex, race, body mass index, waist circumference, lipid-lowering drugs use, hypoglycemic medications use, diabetes, and adiposity

*HR* hazard ratio, *CI* confidence interval

The results of sensitivity analyses are shown in Supplementary Table 8. The association between PsRS and EH risk remained robust after excluding patients diagnosed with EH during the first five years of follow-up or participants who were obese at baseline. Furthermore, after including physical inactivity and current/past smoking as components of PsRS, the effect estimates of the relationship between PsRS and EH incidence were similar.

As shown in Supplementary Fig. 3 A, the relationship between PsRS and the incidence of EH tended to be non-linear (*P* for non-linearity = 0.022). The risk of incident EH gradually increased with PsRS when the PsRS was between 0 and 5 and tended to be stable between 5 and 7. However, when the PsRS was greater than 7, the EH risk began to increase rapidly. Similarly, the same non-linear relationship between PsRS and incident EH risk can be observed in both males and females (*P* for non-linearity < 0.001, Supplementary Fig. 3 C), and males had a higher risk of EH than females even with the same PsRS (*P* for interaction < 0.001).

### Healthy lifestyle score and incident EH

As shown in Fig. 1B, a favorable lifestyle is linked to a lower risk of EH (*P* < 0.001). The 5-year cumulative incidence rates per 1,000 person-years for unfavorable, intermediate, and favorable lifestyle scores were 4.40, 5.66, and 8.05, respectively. Men had higher EH risks than women with the same lifestyle scores (Fig. 1D, Supplementary Table 9). Table 2 indicates that lower healthy lifestyle scores are associated with increased EH risk, with HRs of 0.73 for intermediate and 0.57 for high scores in the unadjusted model. In the fully adjusted model, HRs were 0.86 and 0.77 for intermediate and favorable lifestyles, respectively. Each decrement in lifestyle score was associated with a 14% reduced risk of EH (P for trend < 0.001). The stability of these results was confirmed in sensitivity analyses (Supplementary Table 10) and subgroup analyses (Supplementary Fig. 4).

As shown in Supplementary Fig. 3B, a non-linear relationship was observed between the lifestyle score and EH risk (*P* for non-linearity = 0.038), showing a trend of rapid increase and then a gradual decrease. This finding indicates that the higher the healthy lifestyle scores, the lower the EH risk. Similarly, this non-linear trend can be observed in both males and females (*P* for non-linearity < 0.001, Supplementary Fig. 3D), with men having a higher EH risk than women with the same healthy lifestyle scores (*P* for interaction < 0.001).

### Joint analysis of PsRS and healthy lifestyle score with EH event

**Fig. 2:**
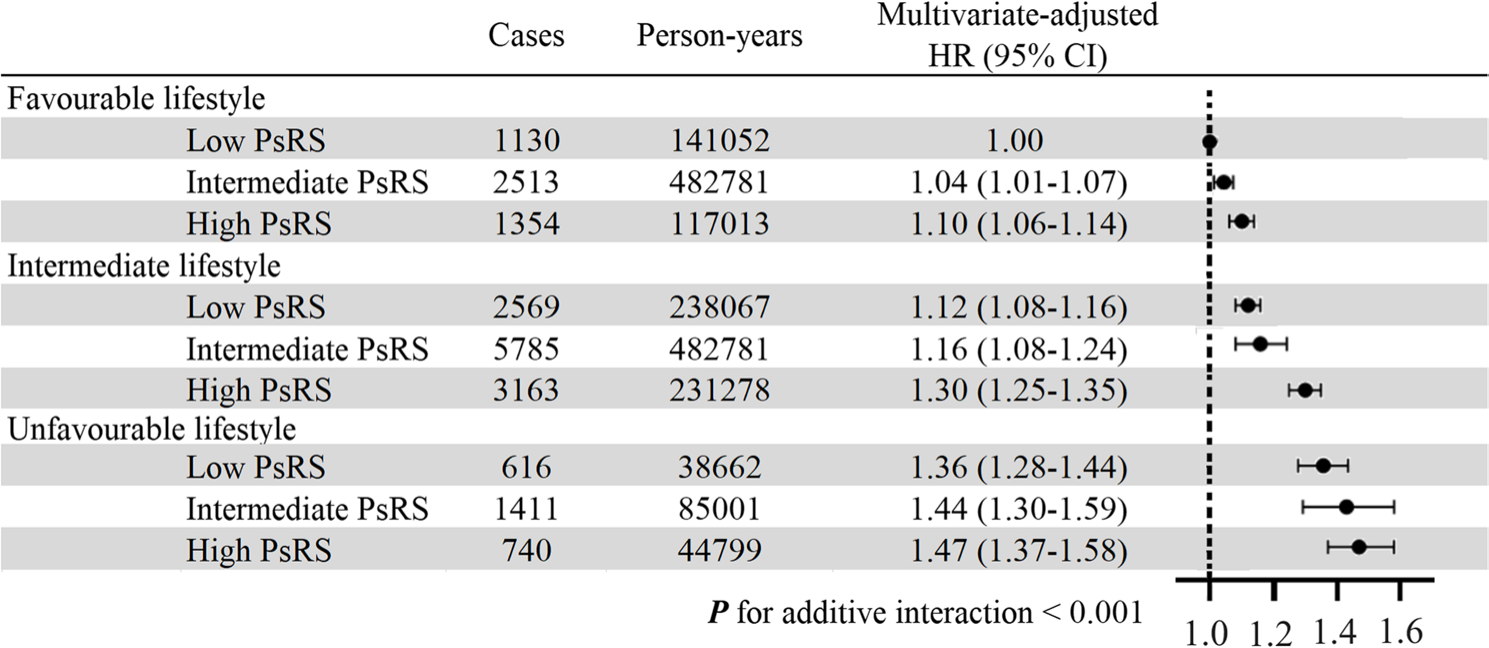
Additive interaction effects of PsRS and lifestyle score on the risk of incident essential hypertension PsRS, polysocial risk score; HR, hazard ratio; CI, confidence interval

The additive interaction effects of PsRS and lifestyle score on the risk of EH are shown in Fig. 2. By using participants with low PsRS and favourable lifestyle score as a reference, participants with higher PsRS or worse lifestyle score generally had an increased risk of EH. Compared with the reference group, the highest risk of EH was observed among people with high PsRS and unfavourable lifestyle scores, with HR being 1.47 (95% CI: 1.37–1.58). All RERIs, APs, and SIs were significant, indicating positive additive interactions between PsRS and lifestyle score on the risk of EH. For example, the SI for high lifestyle score and unfavourable lifestyle score was 2.73 (95% CI: 1.62–3.84), indicating that there was a synergy between social vulnerability and lifestyle; the RERI was 0.47 (95% CI: 0.41–0.54) for participants who had high PsRS and unfavourable lifestyle score, suggesting that there would be 0.47 relative excess risk due to this additive interaction, accounting for 66% (95% CI: 0.54–0.78) of EH risk in those who had high PsRS and unfavourable lifestyle score (Table 3). We also observed significant multiplicative interactions between PsRS and lifestyle scores on EH (*P* for interaction < 0.001, Supplementary Table 11). The mediating effect of lifestyle scores on EH risk across different PsRS groups was relatively low and did not demonstrate significant influence (Supplementary Table 12).

**Table 3:** RERIS, attributable proportions, and synergy indexes for additive interaction between polysocial risk score and healthy lifestyle score on essential hypertension

Categories		RERI (95% CI)	AP (95% CI)	SI (95% CI)
Unfavourable lifestyle				
	High PsRS	0.47 (0.41–0.54)	0.66 (0.54–0.78)	2.73 (1.62–3.84)
	Intermediate PsRS	0.29 (0.23–0.36)	0.51 (0.36–0.66)	1.59 (1.55–1.64)
Intermediate lifestyle				
	High PsRS	0.19 (0.11–0.27)	0.18 (0.10–0.25)	1.37 (1.32–1.44)
	Intermediate PsRS	0.09 (0.02–0.16)	0.11 (0.03–0.18)	1.08 (1.03–1.13)

The models use additional interactions between low PsRS and favourable lifestyle scores as control, and adjusted for age, sex, race, body mass index, waist circumference, lipid-lowering drugs use, hypoglycemic medications use, diabetes, and adiposity

*RERI* Relative excess risk due to the interaction, *AP* Attributable proportions, *SI* Synergy index, *PsRS* Polysocial risk score, *CI* Confidence interval

## Discussion

In this cohort study involving 131,154 participants, a PsRS system was developed by calculating 14 social determinants of socioeconomic status from three aspects, and PsRS was significantly associated with a disproportionately higher risk of incident EH. Our results show that the greater the social vulnerability, the higher the risk of incident EH. Moreover, an unhealthy lifestyle plays an important role in incident EH. The interaction between PsRS and healthy lifestyle scores implies that unhealthy behavioral lifestyles may amplify the adverse effects of social vulnerability on EH. The sex-specific analysis indicated that the influence of social risk factors and behavioral lifestyle on EH events in males is much greater than that in females.

The potential mechanisms underlying the associations between social vulnerability and EH incidence have not been fully understood. However, higher PsRS correlates with greater social vulnerability and increased EH risk, supporting the notion that social vulnerability elevates EH risk [14]. Factors such as family income and education impact living conditions, community exposure, and access to healthcare, affecting EH risk [21]. Improved disease awareness and educational reforms can also reduce EH and cardiovascular disease mortality [22]. Social vulnerability often entails insufficient health knowledge and limited access to health information.

Social vulnerability can be described in the socio-economic aspect, and it plays an important role in social psychology. For example, the existence of emotional distress, such as stress and tension, the care and greetings from relatives and friends, and the existence of mental disorders such as anxiety and depression, are significantly correlated with the risk of EH. Work stress is a common factor for hypertension in modern society [23]. Negative emotional factors such as anxiety, tension, and pressure promote the secretion of various hormones in the body, causing rapid heartbeat, increased cardiac output, and blood vessel contraction. Thus, greater social vulnerability is often associated with a lack of self-psychosomatic regulation and a poorer tolerance for psychological stress [24]. Elevated blood pressure caused by psychosocial stress is an acute stress response that is transient and returns to its original level shortly after the stress is eliminated [25]. However, if the acute stress response is prolonged, blood vessels will remain constricted in response to the stress, further leading to hypertension [26]. The risk of EH may be reduced if the body and mind are relieved by proper relaxation, such as releasing stress through exercise or talking with relatives and friends.

Our study also shows that social and living environments, such as physical deprivation, poor housing quality, and distant green and natural spaces, are significantly associated with incident EH, suggesting that social environmental exposure could be an important risk factor for EH [26]. Long-term exposure to air pollution, cold temperatures, and noise increases the risk of EH in adults. This condition may result from the entry of contaminated air into the bloodstream through the lungs, while noise exposure-associated EH may result from decreased sleep quality, especially under noise exposure at night [27, 28]. Moreover, beneficial effects of the built environment, such as living in a walkable environment or close to green spaces, are associated with a low risk of EH [29]. Therefore, a comfortable living environment may result in good sleep and a happy mood, reduce negative emotions and mental tension, and thus reduce the risk of EH.

A favorable lifestyle lowers the risk of EH [30]. Practices like quitting smoking, limiting alcohol, reducing salt intake, managing weight, and exercising regularly prevent EH and enhance treatment effectiveness [31]. High BMI, especially excess visceral fat, is a major risk factor for EH due to mechanisms such as renal compression and increased sympathetic activity from the renin-angiotensin-aldosterone system [11]. Weight loss in overweight and obese individuals reduces EH risk by 24–54% [32]. Regular physical activity and adherence to a healthy diet, like the DASH diet, significantly lower EH risk [12, 13]. Conversely, heavy alcohol consumption and both insufficient (≤ 5 h) and excessive sleep (≥ 9 h) increase EH risk [33, 34]. Disruptions in sleep patterns from modern life can also contribute to EH, highlighting the need for lifestyle modifications.

This study indicates that behavioral lifestyle factors and social vulnerability exert a significant synergistic effect on the risk of EH. This relationship may be partly explained by the influence of social vulnerability on lifestyle behaviors. Prior studies have established a link between psychosocial stress and increased obesity [35]. Chronic activation of the hypothalamic–pituitary–adrenal axis triggered by stress may promote abdominal fat accumulation through elevated cortisol secretion [36]. Moreover, stress can adversely affect health outcomes by inducing unhealthy behaviors, such as overeating and consumption of high-calorie foods [37]. Higher levels of social stress increases the risk of EH not only through lower physical activity, but also via impaired sleep quality [38]. Residential environment and community facilities, such as neighbouring green spaces, recreational facilities, and street design, play a critical role in shaping residents’ daily behaviour and health status [29]. The synergistic effect of PsRS and lifestyle on EH may also be explained by the fact that an unhealthy lifestyle can exacerbate social vulnerability. For instance, obesity resulting from irregular eating patterns, overeating, and physical inactivity may impose social and psychological stresses on individuals. Some studies suggest that obese people experience greater emotional distress and social isolation compared to those with normal weight [39]. In addition, unhealthy behaviors are not only directly detrimental to human health, but can also negatively impact the living environment. For example, smoking, as a common source of air pollution, releases harmful vapours that contribute to secondhand smoke and adversely affect community health. Higher smoking prevalence within a neighbourhood correlates with an higher risk of EH [40]. Overall, these findings imply that lifestyle interventions alone may not sufficiently reduce EH risk in individuals experiencing high social vulnerability. Complementary strategies targeting social determinants should be integrated into prevention efforts to achieve more effective risk reduction.

Previous evidence has shown that complex interventions targeting social vulnerability had positive effects on function, cognition, subjective health, and reduced hospital utilization [41]. These complex interventions included strengthening social supports and communities, helping older adults and their caregivers navigate health and social services, enhancing neighbourhood and built environments, promoting education and providing economic stability. However, the choice to lump interventions instead of splitting the complex interventions means that the question of which type of intervention, for which populations, or which components of the interventions are most effective remains unanswerable. If the social needs of patients are to be better met in a clinical setting, it is important to consider healthcare providers’ perceptions of the importance of SDOH and strategies to address it [42]. Healthcare providers can take action at the community/societal level, practice/institutional level, and patient level. At the community level, providers are well-positioned to support their patients and advocate for policy and system-level changes that address the upstream social causes of health disparities [43]. At the healthcare institutions and patient level, interventions that address patients’ social needs range from passive data collection and referral services to direct interventions, such as providing on-site services such as legal advice and food pantries [44]. However, poor SDOH status stems from systemic problems that require systems-level solutions. And these solutions go far beyond the healthcare sector and what clinicians can do for their own patients. Therefore, addressing these issues requires a multisectoral approach with strong coordination between healthcare institutions and community-based organizations with a clear focus on SDOH [45]. Healthcare providers with a clear understanding of SDOH can improve health outcomes by helping build such collaborations as well as advocating for policy and systems changes that address the root causes of social problems.

Stratified analysis by sex showed that social risk factors and behavioral lifestyles have more effect on EH events in males than in females. Men tend to face more stress, such as job and economic stress, leading to a higher degree of sympathetic nervous system activation and thus a higher risk of EH [25]. Thus, the prevention and control of EH requires sex-specific measures. With the development of society, some risk factors related to EH, such as unhealthy lifestyle, air pollution, widening wealth gap, and increasing unemployment rate, are constantly changing with great uncertainty. Therefore, active and effective measures should be implemented to reduce the occurrence of EH, such as encouraging education, improving the employment rate, promoting healthy lifestyle, recommending healthy diet patterns, regular health monitoring, and improving the living environment.

This study was the first to comprehensively explore the joint associations of social vulnerability and lifestyles on EH. As a prospective study, this study provides new and strong evidence for the modifiable factors related to the occurrence of EH. However, several limitations should be acknowledged. Firstly, data on lifestyle and social determinants were self-reported and assessed only at enrollment, indicating that we did not take into account potential changes in these factors during follow-up. Further rigorous longitude studies are needed to investigate the dynamic changes in lifestyle factors and socioeconomic status on the risk of EH. Secondly, the UK Biobank participants are generally wealthier than those in developing countries, potentially limiting generalizability. While the study covers more factors than previous research, it does not address genetic susceptibility. Lastly, the PsRS and lifestyle scores assumed equal effects of each component on health, which may not accurately reflect their true impact.

## Conclusions

In conclusion, this prospective study provides new evidence for the joint effect of social determinants of health and behavioral lifestyles on the risk of EH. Greater social vulnerability contributes to a higher risk of EH, and adverse lifestyle plays an important role in the occurrence of EH. An unhealthy lifestyle may amplify the detrimental effect of social vulnerability on EH incidence. Our findings highlight the potential to reduce the risk of EH by modifying these two risk factors individually and simultaneously.

## Supplementary Information

Supplementary Material 1

## Data Availability

The data of the UK Biobank is open to the world, and researchers who apply online will be approved by a dedicated biobank staff before being allowed to use the database. All of our data was extracted directly from the UK Biobank, which is available at www.ukbiobank.ac.uk. This research has been conducted using the UK Biobank Resource under Application Number 97101.
