# Supplementary material for: Joint association of polysocial risk score and lifestyle with incident essential hypertension: a prospective cohort study in the UK biobank
Source: BMC Cardiovasc Disord. 2025 Jul 4;25:460. doi: 10.1186/s12872-025-04930-2 (PMC12231675; doi:10.1186/s12872-025-04930-2)
Supplement: Supplementary file 1 — Supplementary Material 1 [file 12872_2025_4930_MOESM1_ESM.doc]

**Supplementary information**

**Joint association of** **polysocial risk score and lifestyle with incident** **essential hypertension: a prospective cohort study in the UK Biobank**

**Contents**

Supplementary materials (Calculation of PsRS and assessment of healthy lifestyle score)

Supplementary Figure 1. The flow chart of UK Biobank participants for this study.

Supplementary Figure 2. The associations of PsRS with incident essential hypertension stratified by different levels of lifestyle scores.

Supplementary Figure 3. Associations among PsRS (A), lifestyle score (B), sex-specific PsRS (C), sex-specific lifestyle score (D), and incident essential hypertension.

Supplementary Figure 4. The associations of lifestyle score with incident essential hypertension stratified by different levels of PsRS.

Supplementary Table 1. Components of the polysocial risk score and their definitions.

Supplementary Table 2. Associations of individual social determinant of health with incidence of essential hypertension.

Supplementary Table 3. Collinearity test for variables.

Supplementary Table 4. Collinearity test of polysocial risk score.

Supplementary Table 5.Pairwise correlation analysis of multiple polysocial risk score.

Supplementary Table 6. Associations of healthy behavioral lifestyle with incidence of essential hypertension.

Supplementary Table 7. Association of polysocial risk score with incident essential hypertension by sex stratification.

Supplementary Table 8. Sensitivity analysis of the association of polysocial risk score with incidence of essential hypertension.

Supplementary Table 9. Association of healthy lifestyle score with incident essential hypertension by sex stratification.

Supplementary Table 10. Sensitivity analysis of the association of healthy lifestyle score with incidence of essential hypertension.

Supplementary Table 11. The effects of multiplicative interaction between polysocial risk score and healthy lifestyle score on the risk of incident essential hypertension.

Supplementary Table 12. The mediating role of healthy lifestyle score between polysocial risk score and the risk of essential hypertension.

Supplementary Table 13. The additive interaction between multiple polysocial risk score and healthy lifestyle score on the risk of essential hypertension.

**Calculation of PsRS**

PsRS has been identified as a convenient but informative tool for identifying socially vulnerable groups and has been proven to be significantly associated with the risk of type 2 diabetes and cardiovascular diseases (2). Based on the 2030 Healthy Population Initiative guidelines and previous studies, 17 social determinants were pre-selected in this study, which was further divided into three categories, namely, socio-economic situation, social psychological factors, and social environmental conditions (1, 3, 4). For these 17 social determinants, the participants were included in the high-risk group based on the following criteria: (1) gross family income below 31,000 pounds (low income); (2) the degree is lower than college degree (low degree); (3) the score of education quality is lower than the median (poor quality of education); (4) not employed or self-employed; (5) living alone; (6) confide in those around you less than once a week (no social support); (7) less than one group activity per week (lack of social activities); (8) visit relatives and friends less than once a week (social isolation); (9) disease, wounded, bereavement, or pressure (emotional distress) within 2 years; (10) have been diagnosed with a mental disorder, including bipolar disorder, depression and anxiety, even have autotomy (mental disturbance); (11) Townsend deprivation index (TDI) scores higher than the median; (12) the neighborhood has a higher than median crime score (high local crime rate); (13) housing scores higher than the median (bad housing); (14) house not owned (unstable accommodation); and (15–17) percentage of home location buffers classified as green space (greenbelt remote), water (blue space remote), and natural environment (bad natural) is under median. The description and UK Biobank data code of these 17 social determinants are shown in Supplementary Table 1. Since it is necessary to conduct statistical tests on multiple social determinants in the same dataset, the Bonferroni method was used in this study to correct *P* values and reduce the probability of Type Ⅰ errors. In other words, each social determinant of health was treated as a binary variable to determine whether participants were exposed. If the social determinants were significantly related to EH event with a Bonferroni's corrected *P* values < 0.003 (0.05/17 comparisons) in a fully adjusted Cox regression analysis with adjustment for age, sex, race, body mass index (BMI), smoking status, alcohol intake, physical activity, diet quality score, and sleep quality score, they will be incorporated into the calculation of PsRS. The higher the PsRS, the greater the social vulnerability.

**Assessment of healthy lifestyle score**

Based on previous literature, a healthy lifestyle score was set according to five behavioral lifestyle factors, namely, smoking status, alcohol consumption, sports activities, food, and sleep quality (5). Weekly alcohol consumption was assessed using a baseline touch-screen questionnaire based on the frequency of alcohol consumption. Sports activity was evaluated according to the persistent time and frequency of tramping, modest activity, and drastic activity. Diet was evaluated by calculating the diet quality score based on their intake of greens, fruits, whole grains, fish, processed meat, refined grains, and mammalian meat. One point was assigned to the food item if the following average intake: (1) fruits ≥ 3 servings/day; (2) vegetables ≥ 3 servings/day; (3) fish ≥ 2 servings/week; (4) processed meat ≤ 1 servings/week; (5) red meat ≤ 1.5 servings/week; (6) whole grain ≥ 3 servings/week; (7) refined grain ≤ 1.5 servings/week. The diet quality score ranged from 0 to 7 with a higher score indicating a better diet quality. Sleep quality was scored based on sleep type, sleep duration, frequency of insomnia, snoring, and daytime sleepiness. One point was assigned to each item based on the following criteria: (1) early chronotype; (2) 7–8 hours of sleep per day; (3) never or rarely had insomnia symptoms; (4) no self-reported snoring; and (5) no frequent daytime sleepiness. The sleep quality score ranged from 0 to 5 with a higher score indicating a healthier sleep pattern.

For healthy lifestyle scores, participants were given one point if they met one of the following criteria: (1) never smoked; (2) alcohol consumption in moderation or not at all (drink < 3 times/week); (3) enough physical activity (satisfy any one criteria: modest activity ≥ 5 times/week or ≥ 150 min/week, or vigorous activity ≥ 1 time/week or ≥ 75 min/ week, or ≥ 150 min/week of combined moderate and vigorous activity); (4) healthy diet (diet score ≥ 4); and (5) healthy slumber (slumber score ≥ 4). Healthy lifestyle scores ranged from 0 to 5, with a higher score indicating a better or ideal lifestyle. The healthy lifestyle scores were divided into three groups, namely, unfavorable (0−1), intermediate (2−3), and favorable (4−5).


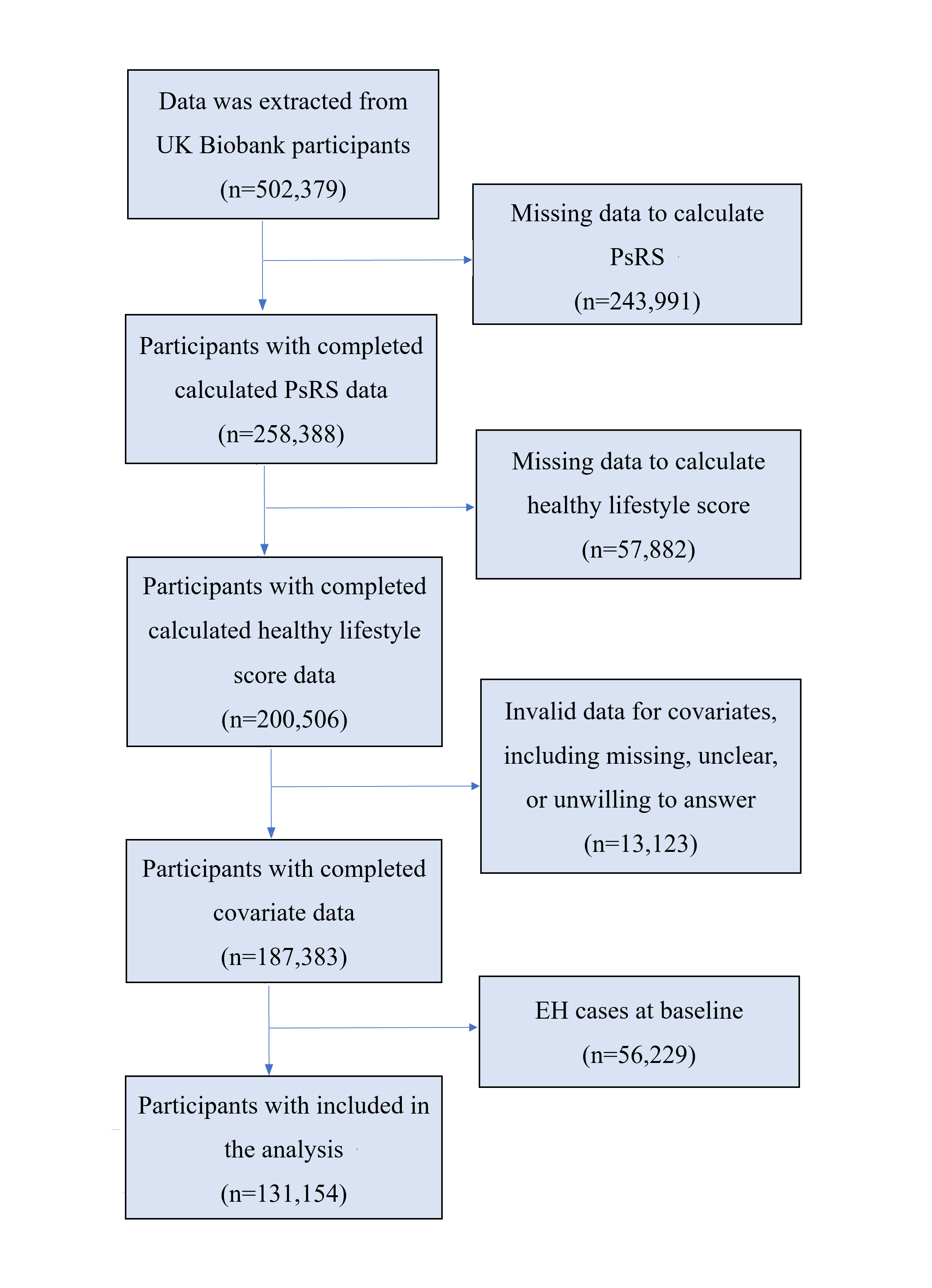


**Supplementary Figure 1.** The flow chart of UK Biobank participants for this study.

PsRS, polysocial risk score; EH, essential hypertension.


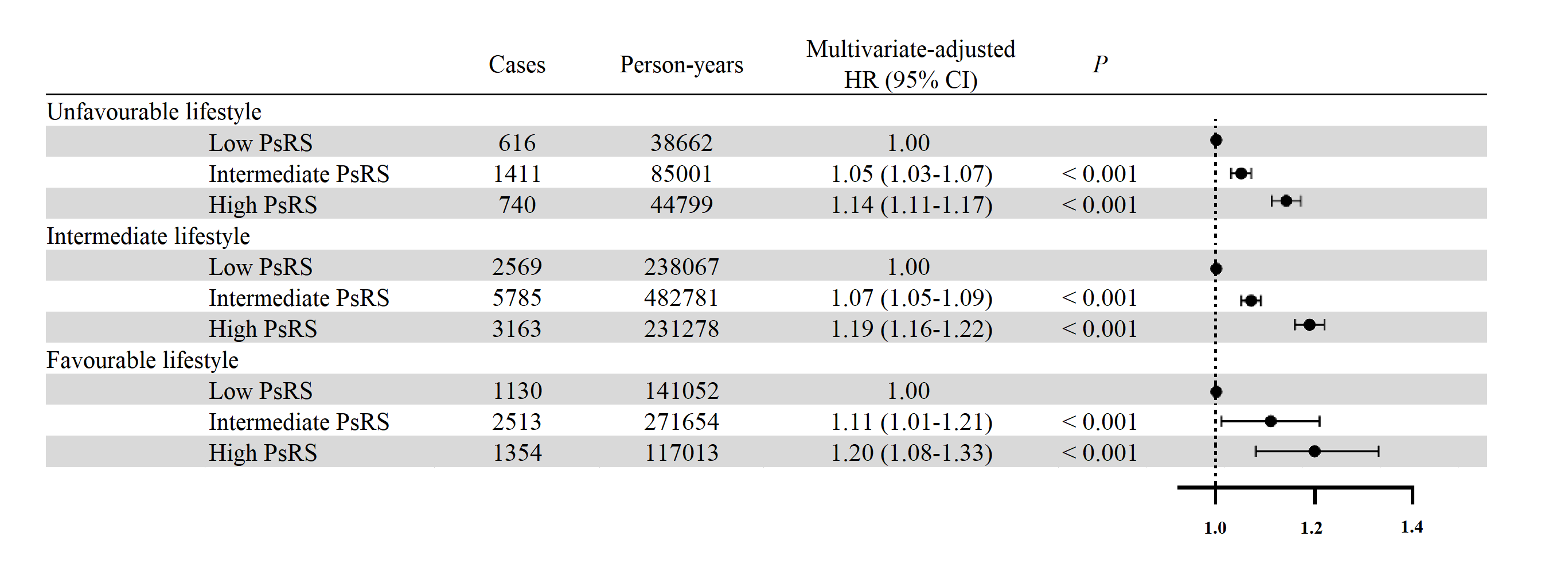


**Supplementary Figure 2.** The associations of PsRS with incident essential hypertension stratified by different levels of lifestyle scores.

PsRS, polysocial risk score; HR, hazard ratio; CI, confidence interval.


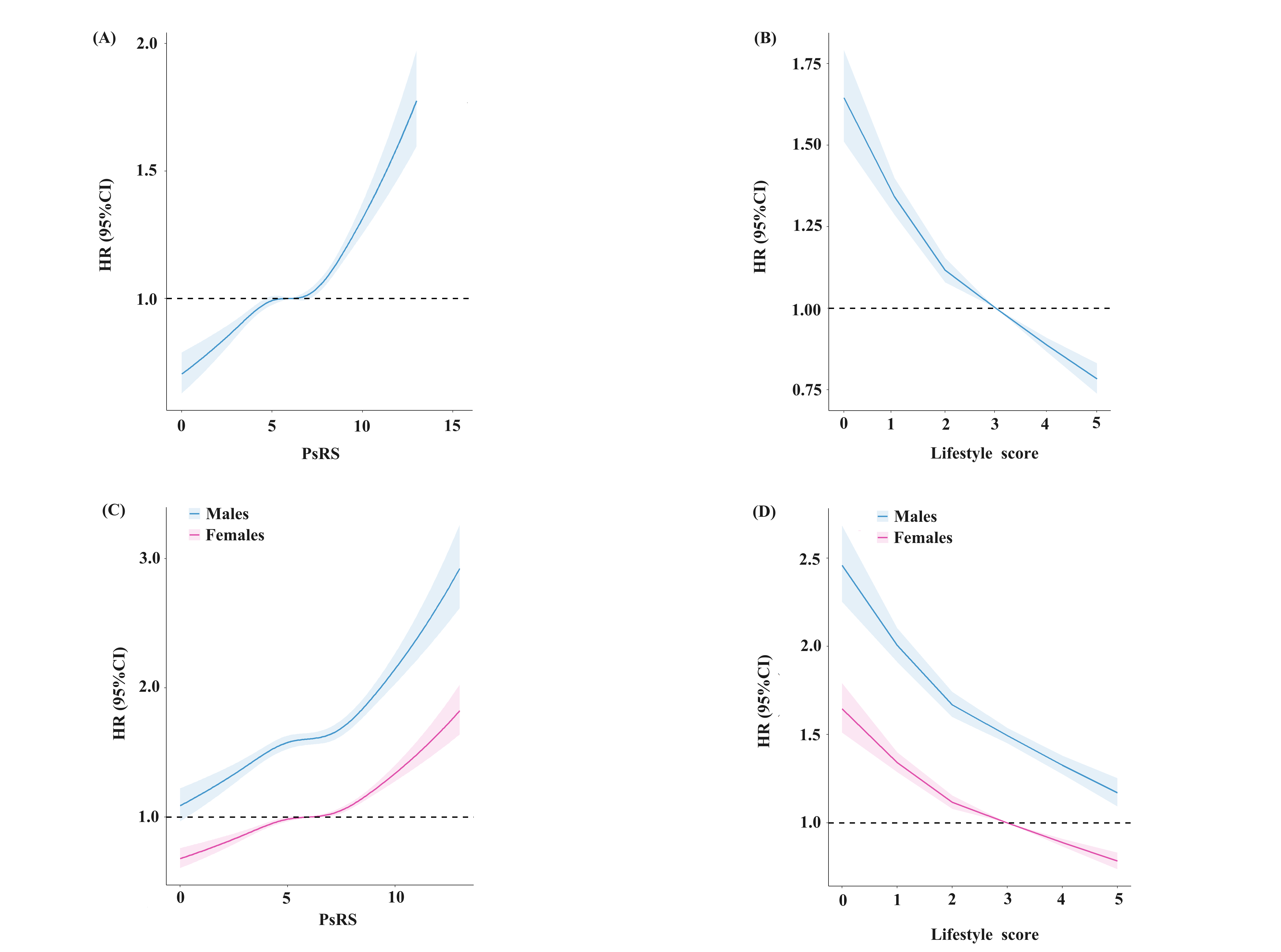


**Supplementary Figure 3.** Associations among PsRS (A), lifestyle score (B), sex-specific PsRS (C), sex-specific lifestyle score (D), and incident essential hypertension.

PsRS, polysocial risk score; HR, hazard ratio; CI, confidence interval.


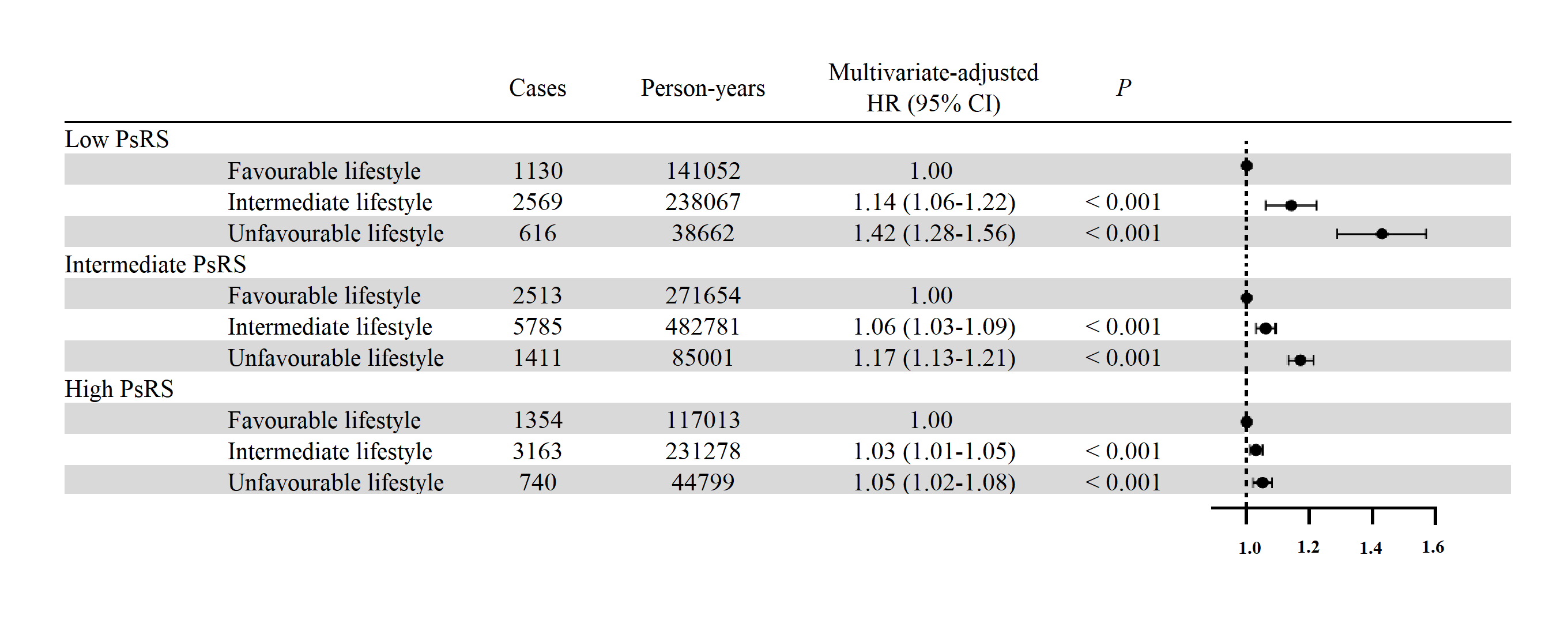


**Supplementary Figure 4.** The associations of lifestyle score with incident essential hypertension stratified by different levels of PsRS.

PsRS, polysocial risk score; HR, hazard ratio; CI, confidence interval.

**Supplementary Table 1: Components of the polysocial risk score and their definitions.**

| **Social determinant of health** | **Description (1 = at-risk, 0 = reference)** | **UK Biobank data code** |
| --- | --- | --- |
| Socioeconomic status |  |  |
| Low household income | 1 = less than £31,000; 0 = average total household income before tax is more than £31,000 | 738 |
| Low degree | 1 = lower than college; 0 = highest education level is college or above | 6138 |
| Poor quality of education | 1 = above the median of education score; 0 = below the median | 26414, 26431, and 26421 |
| Not employment | 0 = In paid employment or self-employed; 1 = others | 6142 |
| Social psychosocial factors |  |  |
| Living alone | 1= yes; 0 = no | 709 |
| Lack of social support | 1 = once a week or less often; 0 = able to confide in anyone close to you at least once a week | 2110 |
| lack of social activities | 1 = less often than once a week; 0 = attend any group activities once a week or more often | 6160 |
| Social isolation | 1 = once a week or less often; 0 = visit friend/family or have them visit you more often than once a week | 1031 |
| Emotional distress | 1 = have experienced illness, injury, bereavement, stress within last 2 years; 0 = none | 6145 |
| Diagnosed mental disorder | 1 = with diagnosed psychiatric disorders including anxiety, depression, and bipolar disorder, or ever self-harmed; 0 = none | 20002, 20544, 20126, 20480, and 20485 |
| Social environment condition | |  |
| Area-level material deprivation | 1 = above the median of Townsend deprivation index; 0 = below the median | 22189 |
| High local crime rate | 1 = above the median of crime score; 0 = below the median | 26416, 26434, and 26425 |
| Poor housing quality | 1 = above the median of housing score; 0 = below the median | 26415, 26432, and 26423 |
| Unstable accommodation | 0 = own current accommodation outright; 1 = own with mortgage | 680 |
| Greenspace remote | 0 = above the median of percentage of home location buffer classed as greenspace; 1 = below the median | 24500 |
| Blue space remote | 0 = above the median of percentage of home location buffer classed as water; 1 = below the median | 24502 |
| Natural environment remote | 0 = above the median of percentage of home location buffer classed as natural land; 1 = below the median | 24506 |

**Supplementary Table 2: Associations of individual social determinant of health with incidence of essential hypertension.**

| Social determinant of health | Participants at risk, % | Fully adjusted HR (95% CI) | *P* value |
| --- | --- | --- | --- |
| Socioeconomic status | | | |
| Low household income | 36.00% | 1.26 (1.20-1.31) | < 0.0001* |
| Low education degree | 55.40% | 1.22 (1.09-1.15) | < 0.0001* |
| Poor quality of education | 58.20% | 1.31 (1.21-1.42) | < 0.0001* |
| Not in paid employment | 31.60% | 1.18 (1.12-1.24) | 0.0021* |
| Social psychosocial factors | | | |
| Living alone | 14.90% | 1.02 (0.94-1.10) | 0.3856 |
| Lack of social support | 33.70% | 1.29 (1.18-1.40) | < 0.0001* |
| lack of social activities | 27.80% | 1.01 (0.92-1.10) | 0.8174 |
| Social isolation | 23.00% | 1.16 (1.07-1.25) | 0.0027* |
| Emotional distress | 44.40% | 1.41 (1.26-1.56) | < 0.0001* |
| Diagnosed mental disorder | 27.10% | 1.20 (1.12-1.28) | 0.0015* |
| Social environment condition | | | |
| Area-level material deprivation | 46.00% | 1.17 (1.13-1.21) | 0.0019* |
| High local crime rate | 47.00% | 1.33 (1.20-1.46) | < 0.0001* |
| Poor housing quality | 47.80% | 1.17 (1.12-1.22) | 0.0020* |
| Unstable accommodation | 51.40% | 1.34 (1.27-1.41) | < 0.0001* |
| Greenspace remote | 50.00% | 1.24 (1.19-1.30) | < 0.0001* |
| Blue space remote | 50.80% | 0.99 (0.93-1.05) | 0.4685 |
| Natural environment remote | 48.80% | 1.26 (1.18-1.34) | < 0.0001* |

Note: The HRs and 95% CI were calculated using a fully adjusted Cox hazard model with adjustment for age, sex, race, body mass index, smoking status, alcohol intake, physical activity, diet quality score, and sleep quality score.

Abbreviations: HR, hazard ratio; CI, confidence interval. Social determinants of health with a * marker was included in the calculation of the PsRS.

**Supplementary Table 3. Collinearity test for variables.**

| Variable | VIF |
| --- | --- |
| Polysocial risk score | 1.012 |
| Healthy lifestyle score | 2.456 |
| Age | 1.046 |
| Sex | 1.381 |
| Race | 1.008 |
| Body mass index | 2.217 |
| Waist circumferenc | 2.384 |
| Smoking status | 1.027 |
| Alcohol intake | 1.045 |
| Sleep quality score | 1.005 |
| Lipid-lowering drugs use | 1.094 |
| Hypoglycemic medications use | 1.014 |
| Diabetes | 1.119 |
| Adiposity | 1.125 |
| Physical activity | 1.129 |
| Diet quality score | 1.525 |

**Supplementary Table 4. Collinearity test of polysocial risk score.**

| Social determinant of health | VIF |
| --- | --- |
| Socioeconomic status |  |
| Low household income | 1.360 |
| Low education degree | 1.099 |
| Poor quality of education | 1.270 |
| Not in paid employment | 1.327 |
| Social psychosocial factors |  |
| Living alone | 1.127 |
| Lack of social support | 1.038 |
| lack of social activities | 1.018 |
| Social isolation | 1.029 |
| Emotional distress | 1.030 |
| Diagnosed mental disorder | 1.013 |
| Social environment condition |  |
| Area-level material deprivation | 1.357 |
| High local crime rate | 1.288 |
| Poor housing quality | 1.037 |
| Unstable accommodation | 1.225 |
| Greenspace remote | 3.993 |
| Blue space remote | 1.024 |
| Natural environment remote | 4.042 |

**Supplementary Table 5.Pairwise correlation analysis of multiple polysocial risk score**.

|  | Low household income | Low education degree | Poor quality of education | Not in paid employment | Lack of social support | Social isolation | Emotional distress | Diagnosed mental disorder | Area-level material deprivation | High local crime rate | Poor housing quality | Unstable accommodation | Greenspace remote | Natural environment remote | Living alone | lack of social activities | Blue space remote |
| --- | --- | --- | --- | --- | --- | --- | --- | --- | --- | --- | --- | --- | --- | --- | --- | --- | --- |
| Low household income | 1 | 0.221 | -0.183 | 0.346 | 0.082 | -0.073 | 0.066 | 0.015 | 0.095 | 0.066 | 0.069 | -0.172 | 0.01 | 0.017 | 0.259 | -0.11 | 0.008 |
| Low education degree | 0.221 | 1 | -0.179 | 0.07 | 0.03 | -0.087 | 0.036 | -0.038 | -0.027 | -0.008 | 0.089 | 0.01 | -0.07 | -0.059 | -0.001 | -0.029 | 0.016 |
| Poor quality of education | -0.183 | -0.179 | 1 | 0.048 | -0.032 | 0.025 | -0.064 | -0.014 | -0.294 | -0.297 | -0.096 | -0.099 | -0.034 | -0.06 | -0.084 | 0.043 | -0.02 |
| Not in paid employment | 0.346 | 0.07 | 0.048 | 1 | 0.023 | -0.091 | -0.03 | -0.027 | -0.057 | -0.051 | 0.011 | -0.367 | -0.044 | -0.051 | 0.045 | -0.085 | -0.006 |
| Lack of social support | 0.082 | 0.03 | -0.032 | 0.023 | 1 | 0.079 | 0.022 | 0.025 | 0.029 | 0.024 | -0.007 | 0.003 | 0.017 | 0.02 | 0.149 | -0.003 | 0.004 |
| Social isolation | -0.073 | -0.087 | 0.025 | -0.091 | 0.079 | 1 | -0.024 | 0.014 | 0.019 | 0.012 | -0.037 | 0.055 | 0.02 | 0.019 | -0.041 | 0.044 | -0.007 |
| Emotional distress | 0.066 | 0.036 | -0.064 | -0.03 | 0.022 | -0.024 | 1 | 0.064 | 0.062 | 0.038 | 0 | 0.11 | 0.031 | 0.035 | 0.043 | -0.014 | 0.005 |
| Diagnosed mental disorder | 0.015 | -0.038 | -0.014 | -0.027 | 0.025 | 0.014 | 0.064 | 1 | 0.04 | 0.027 | -0.013 | 0.05 | 0.033 | 0.03 | 0.056 | -0.002 | -0.001 |
| Area-level material deprivation | 0.095 | -0.027 | -0.294 | -0.057 | 0.029 | 0.019 | 0.062 | 0.04 | 1 | 0.376 | -0.088 | 0.108 | 0.332 | 0.343 | 0.148 | -0.027 | 0.008 |
| High local crime rate | 0.066 | -0.008 | -0.297 | -0.051 | 0.024 | 0.012 | 0.038 | 0.027 | 0.376 | 1 | -0.065 | 0.076 | 0.295 | 0.304 | 0.083 | -0.017 | 0.064 |
| Poor housing quality | 0.069 | 0.089 | -0.096 | 0.011 | -0.007 | -0.037 | 0 | -0.013 | -0.088 | -0.065 | 1 | -0.01 | -0.007 | -0.017 | -0.024 | -0.012 | 0.005 |
| Unstable accommodation | -0.172 | 0.01 | -0.099 | -0.367 | 0.003 | 0.055 | 0.11 | 0.05 | 0.108 | 0.076 | -0.01 | 1 | 0.055 | 0.062 | 0.006 | 0.057 | 0.002 |
| Greenspace remote | 0.01 | -0.07 | -0.034 | -0.044 | 0.017 | 0.02 | 0.031 | 0.033 | 0.332 | 0.295 | -0.007 | 0.055 | 1 | 0.865 | 0.08 | -0.006 | 0.111 |
| Natural environment remote | 0.017 | -0.059 | -0.06 | -0.051 | 0.02 | 0.019 | 0.035 | 0.03 | 0.343 | 0.304 | -0.017 | 0.062 | 0.865 | 1 | 0.082 | -0.008 | 0.139 |
| Living alone | 0.259 | -0.001 | -0.084 | 0.045 | 0.149 | -0.041 | 0.043 | 0.056 | 0.148 | 0.083 | -0.024 | 0.006 | 0.08 | 0.082 | 1 | -0.035 | -0.009 |
| lack of social activities | -0.11 | -0.029 | 0.043 | -0.085 | -0.003 | 0.044 | -0.014 | -0.002 | -0.027 | -0.017 | -0.012 | 0.057 | -0.006 | -0.008 | -0.035 | 1 | -0.006 |
| Blue space remote | 0.008 | 0.016 | -0.02 | -0.006 | 0.004 | -0.007 | 0.005 | -0.001 | 0.008 | 0.064 | 0.005 | 0.002 | 0.111 | 0.139 | -0.009 | -0.006 | 1 |

**Supplementary Table 6. Associations of healthy behavioral lifestyle with incidence of essential hypertension.**

| Lifestyle | Participants in  healthy lifestyle, % | Fully adjusted HR (95% CI) | *P* value |
| --- | --- | --- | --- |
| Never smoked | 57.30% | 0.62 (0.59 - 0.65) | < 0.001 |
| Drink moderately or not at all ^a^ | 51.40% | 0.85 (0.82 - 0.88) | < 0.001 |
| Enough physical activity ^b^ | 96.30% | 0.69 (0.64 - 0.74) | < 0.001 |
| Healthy diet ^c^ | 46.50% | 0.78 (0.75 - 0.81) | < 0.001 |
| Healthy sleep ^d^ | 40.90% | 0.91 (0.89 - 0.94) | < 0.001 |

Note: The HRs and 95% CI were calculated using a fully adjusted Cox hazard model with adjustment for age, sex, race, and body mass index.

Abbreviations: HR, hazard ratio; CI, confidence interval.

^a^ Drink moderately was defined as less than three times a week;

^b^ Enough physical activity was defined as ≥ 150 min/week of moderate activity, or ≥ 75 min/week of vigorous activity, or ≥ 150 min/week of combined moderate and vigorous activity, ≥ 5 times/week of moderate activity, or ≥ 1 time/week of vigorous activity;

^c^ Healthy diet was defined as diet score ≥ 4;

^d^ Healthy sleep was defined as sleep score ≥ 4.

**Supplementary Table 7. Association of polysocial risk score with incident essential hypertension by sex stratification.**

| Sex stratification | Polysocial risk score | | | | | Per point increment | *P* for trend |
| --- | --- | --- | --- | --- | --- | --- | --- |
|  | Low (≤4) | Intermediate (5-7) | *P* | High (≥8) | *P* |  |  |
| Males |  |  |  |  |  |  |  |
| Cases | 2615 (8.03%) | 5301 (7.94%) |  | 2621 (8.23%) |  |  |  |
| Person-years | 196054 | 362497 |  | 155009 |  |  |  |
| Unadjusted HR (95% CI) | 1.00 | 1.09 (1.04-1.14) | < 0.001 | 1.26 (1.19-1.33) | < 0.001 | 1.12 (1.09-1.15) | < 0.001 |
| Fully adjusted HR (95% CI) | 1.00 | 1.11 (1.06-1.16) | < 0.001 | 1.33 (1.26-1.38) | < 0.001 | 1.16 (1.13-1.18) | < 0.001 |
| Females |  |  |  |  |  |  |  |
| Cases | 1700 (5.22%) | 4408 (6.60%) |  | 2635 (8.28%) |  |  |  |
| Person-years | 221727 | 476940 |  | 238082 |  |  |  |
| Unadjusted HR (95% CI) | 1.00 | 1.19 (1.13-1.26) | < 0.001 | 1.43 (1.34-1.52) | < 0.001 | 1.19 (1.16-1.23) | < 0.001 |
| Fully adjusted HR (95% CI) | 1.00 | 1.12 (1.07-1.18) | < 0.001 | 1.32 (1.25-1.40) | < 0.001 | 1.15 (1.13-1.17) | < 0.001 |

Note: HRs and 95% CIs were calculated using a fully adjusted Cox proportional hazard model with adjustment for age, sex, race, body mass index, waist circumference, smoking status, alcohol intake, physical activity, diet quality score, sleep quality score, lipid-lowering drugs use, hypoglycemic medications use, diabetes, and adiposity.

HR, hazard ratio; CI, confidence interval.

**Supplementary Table 8. Sensitivity analysis of the association of polysocial risk score with incidence of essential hypertension.**

|  | Polysocial risk score | | | | | Per point increment | *P* for trend |
| --- | --- | --- | --- | --- | --- | --- | --- |
|  | Low (≤ 4) | Intermediate (5-7) | *P* value | High (≥ 8) | *P* value |  |  |
| Excluding cases occurred in the first 5 years | | | | | | | |
| Cases | 3015 | 6592 |  | 3573 |  |  |  |
| Person-years | 414332 | 831021 |  | 388553 |  |  |  |
| Unadjusted HR (95% CI) | 1.00 | 1.18 (1.04 - 1.13) | < 0.001 | 1.26 (1.20 - 1.32) | < 0.001 | 1.12 (1.09 - 1.15) | < 0.001 |
| Fully adjusted HR (95% CI) | 1.00 | 1.08 (1.04 - 1.12) | < 0.001 | 1.29 (1.24 - 1.34) | < 0.001 | 1.14 (1.12 - 1.16) | < 0.001 |
| Excluding subjects with obesity at baseline ^a^ | | | | | | | |
| Cases | 2542 | 5460 |  | 2701 |  |  |  |
| Person-years | 304740 | 597538 |  | 265827 |  |  |  |
| Unadjusted HR (95% CI) | 1.00 | 1.09 (1.03 - 1.14) | < 0.001 | 1.21 (1.14 - 1.28) | < 0.001 | 1.10 (1.07 - 1.13) | < 0.001 |
| Fully adjusted HR (95% CI) | 1.00 | 1.11 (1.05 - 1.17) | < 0.001 | 1.25 (1.20 - 1.30) | < 0.001 | 1.13 (1.08 - 1.17) | < 0.001 |
| Including inadequate physical activity and ever/current smoking in calculating PsRS ^b^ | | | | | | | |
| Cases | 2928 | 9399 |  | 6954 |  |  |  |
| Person-years | 297741 | 822053 |  | 530514 |  |  |  |
| Unadjusted HR (95% CI) | 1.00 | 1.16 (1.11 - 1.21) | < 0.001 | 1.32 (1.27 - 1.38) | < 0.001 | 1.15 (1.13 - 1.17) | < 0.001 |
| Fully adjusted HR (95% CI) | 1.00 | 1.13 (1.08 - 1.17) | < 0.001 | 1.30 (1.24 - 1.36) | < 0.001 | 1.13 (1.10 - 1.17) | < 0.001 |
| Exclude subjects with prehypertension |  |  |  |  |  |  |  |
| Cases | 3930 | 8898 |  | 4778 |  |  |  |
| Person-years | 371364 | 749197 |  | 351063 |  |  |  |
| Unadjusted HR (95% CI) | 1.00 | 1.11 (1.07 - 1.16) | < 0.001 | 1.28 (1.22 - 1.33) | < 0.001 | 1.05 (1.04 - 1.06) | < 0.001 |
| Fully adjusted HR (95% CI) | 1.00 | 1.06 (1.02 - 1.10) | 0.001 | 1.16 (1.11 - 1.21) | < 0.001 | 1.03 (1.02 - 1.04) | < 0.001 |

Note: ^a^ HRs and 95% CIs were calculated using a fully adjusted Cox proportional hazard model with adjustment for age, sex, race, body mass index, waist circumference, smoking status, alcohol intake, physical activity, diet quality score, sleep quality score, lipid-lowering drugs use, hypoglycemic medications use, diabetes, and adiposity.

^b^ HRs and 95% CIs were calculated using a fully adjusted Cox proportional hazard model with adjustment for age, sex, race, body mass index, waist circumference, alcohol intake, diet quality score, sleep quality score, lipid-lowering drugs use, hypoglycemic medications use, diabetes, and adiposity.

^a^ Obesity was defined as BMI ≥ 28 kg/m^2^ or males with waist circumference＞85 cm or females with waist＞80 cm.

PsRS, polysocial risk score; HR, hazard ratio; CI, confidence interval.

**Supplementary Table 9. Association of healthy lifestyle score with incident essential hypertension by sex stratification.**

| Sex stratification | Healthy lifestyle score | | | | | Per point decrement | *P* for trend |
| --- | --- | --- | --- | --- | --- | --- | --- |
|  | Unfavourable (0-1) | Intermediate  (2-3) | *P* value | Favourable  (4-5) | *P* value |  |  |
| Males |  |  |  |  |  |  |  |
| Cases | 2064 | 6565 |  | 1909 |  |  |  |
| Person-years | 103965 | 442597 |  | 166997 |  |  |  |
| Unadjusted HR (95% CI) | 1.00 | 0.74 (0.70-0.78) | < 0.001 | 0.57 (0.53-0.61) | < 0.001 | 0.75 (0.73-0.78) | < 0.001 |
| Fully adjusted HR (95% CI) | 1.00 | 0.80 (0.76-0.84) | < 0.001 | 0.65 (0.62-0.69) | < 0.001 | 0.82 (0.80-0.84) | < 0.001 |
| Females |  |  |  |  |  |  |  |
| Cases | 703 | 4952 |  | 3088 |  |  |  |
| Person-years | 64497 | 509529 |  | 362723 |  |  |  |
| Unadjusted HR (95% CI) | 1.00 | 0.89 (0.82-0.96) | < 0.001 | 0.78 (0.71-0.84) | < 0.001 | 0.88 (0.85-0.91) | < 0.001 |
| Fully adjusted HR (95% CI) | 1.00 | 0.91 (0.86-0.96) | < 0.001 | 0.84 (0.76-0.92) | < 0.001 | 0.90 (0.86-0.94) | < 0.001 |

Note: HRs and 95% CIs were calculated using a fully adjusted Cox proportional hazard model with adjustment for age, sex, race, body mass index, waist circumference, lipid-lowering drugs use, hypoglycemic medications use, diabetes, and adiposity.

HR, hazard ratio; CI, confidence interval.

**Supplementary Table 10. Sensitivity analysis of the association of healthy lifestyle score with incidence of essential hypertension.**

|  | Healthy lifestyle score | | | | | Per point increment | *P* for trend |
| --- | --- | --- | --- | --- | --- | --- | --- |
|  | Unfavorable (0-1) | Intermediate  (2-3) | *P* value | Favorable  (4-5) | *P* value |  |  |
| Excluding cases occurred in the first 5 years | | | | | | | |
| Cases | 3015 | 6592 |  | 3573 |  |  |  |
| Person-years | 414332 | 831021 |  | 388553 |  |  |  |
| Unadjusted HR (95% CI) | 1.00 | 0.75 (0.71 - 0.79) | < 0.001 | 0.59 (0.55 - 0.63) | < 0.001 | 0.77 (0.75 - 0.79) | < 0.001 |
| Fully adjusted HR (95% CI) | 1.00 | 0.84 (0.82 - 0.87) | < 0.001 | 0.74 (0.70 - 0.77) | < 0.001 | 0.86 (0.84 - 0.87) | < 0.001 |
| Excluding subjects with obesity at baseline ^a^ | | | | | | | |
| Cases | 2542 | 5460 |  | 2701 |  |  |  |
| Person-years | 304740 | 597538 |  | 265827 |  |  |  |
| Unadjusted HR (95% CI) | 1.00 | 0.71 (0.67 - 0.75) | < 0.001 | 0.56 (0.52 - 0.60) | < 0.001 | 0.75 (0.73 - 0.78) | < 0.001 |
| Fully adjusted HR (95% CI) | 1.00 | 0.80 (0.77 - 0.84) | < 0.001 | 0.71 (0.68 - 0.74) | < 0.001 | 0.85 (0.83 - 0.87) | < 0.001 |
| Exclude subjects with prehypertension |  |  |  |  |  |  |  |
| Cases | 2527 | 10505 |  | 4574 |  |  |  |
| Person-years | 149581 | 846263 |  | 475780 |  |  |  |
| Unadjusted HR (95% CI) | 1.00 | 0.73 (0.70 - 0.77) | < 0.001 | 0.57 (0.54 - 0.60) | < 0.001 | 0.84 (0.83 - 0.85) | < 0.001 |
| Fully adjusted HR (95% CI) | 1.00 | 0.79 (0.76 - 0.83) | < 0.001 | 0.69 (0.65 - 0.73) | < 0.001 | 0.88 (0.87 - 0.90) | < 0.001 |

Note: HRs and 95% CIs were calculated using a fully adjusted Cox proportional hazard model with adjustment for age, sex, race, body mass index, waist circumference, lipid-lowering drugs use, hypoglycemic medications use, diabetes, and adiposity.

^a^ Obesity was defined as BMI ≥ 28 kg/m^2^ or males with waist circumference＞85 cm or females with waist＞80 cm.

HR, hazard ratio; CI, confidence interva

**Supplementary Table 11.** **The effects of multiplicative interaction between polysocial risk score and healthy lifestyle score on the risk of incident essential hypertension.**

| PsRS categories | Lifestyle score (HR, 95% CI) | | | *P* for interaction |
| --- | --- | --- | --- | --- |
|  | Favourable | Intermediate | Unfavourable |  |
| Low | 1.00 |  |  | < 0.001 |
| Intermediate |  | 1.07 (1.03-1.33) | 1.33 (1.25-1.41) |  |
| High |  | 1.21 (1.16-1.26) | 1.42 (1.32-1.52) |  |

Note: HR and 95% CIs were calculated using a multivariate-adjusted Cox proportional hazards model with adjustment for age, sex, race, body mass index, waist circumference, lipid-lowering drugs use, hypoglycemic medications use, diabetes, and adiposity.

HR, hazard ratio; CI, confidence interval.

**Supplementary Table 12. The mediating role of healthy lifestyle score between polysocial risk score and the risk of essential hypertension.**

| PsRS categories | Lifestyle score (Mediation proportion (%),95% CI) | | |
| --- | --- | --- | --- |
|  | Favourable  (N=41,365) | Intermediate  (N=75,898) | Unfavourable  (N=13,891) |
| Low  (N=32,566) | 1 |  |  |
| Intermediate (N=66,754) |  | 1.78% (1.39%-2.44%) | 5.50% (5.02%-6.49%) |
| High  (N=31,834) |  | 1.46% (0.93%-1.66%) | 6.34% (5.80%-7.60%) |

Note: The mediation model has been adjusted for variables such as age, sex, race, body mass index, waist circumference, lipid-lowering drugs use, hypoglycemic medications use, diabetes, and adiposity. CI, confidence interval.

**Supplementary Table 13. The additive interaction between multiple polysocial risk score and healthy lifestyle score on the risk of essential hypertension.**

| PsRS categories | Lifestyle score (HR, 95% CI) | | |
| --- | --- | --- | --- |
|  | Favourable  (N=41,365) | Intermediate  (N=75,898) | Unfavourable  (N=13,891) |
| Low  (N=32,566) | 1 | 1.20 (1.12 - 1.29) | 1.51 (1.37 - 1.68) |
| Intermediate (N=66,754) | 1.10 (1.02 - 1.18) | 1.27 (1.19 - 1.36) | 1.64 (1.51 - 1.79) |
| High  (N=31,834) | 1.28 (1.18 - 1.39) | 1.40 (1.31 - 1.50) | 1.67 (1.52 - 1.84) |

Note: HR and 95% CIs were calculated using a multivariate-adjusted Cox proportional hazards model with adjustment for age, sex, race, body mass index, waist circumference, lipid-lowering drugs use, hypoglycemic medications use, diabetes, and adiposity.

HR, hazard ratio; CI, confidence interval.

**References**

1. Kreuter MW, Thompson T, McQueen A, Garg R. Addressing Social Needs in Health Care Settings: Evidence, Challenges, and Opportunities for Public Health. *Annu Rev Public Health*. (2021);42:329-44.

2. Zhao Y, Li Y, Zhuang Z, Song Z, Wang W, Huang N, et al. Associations of polysocial risk score, lifestyle and genetic factors with incident type 2 diabetes: a prospective cohort study. *Diabetologia*. (2022);65(12):2056-65.

3. Ping Y, Odden MC, Stawski RS, Abdel Magid HS, Wu C. Creation and validation of a polysocial score for mortality among community-dwelling older adults in the USA: the health and retirement study. *Age Ageing*. (2021);50(6):2214-21.

4. Braveman P, Egerter S, Williams DR. The social determinants of health: coming of age. *Annu Rev Public Health*. (2011);32:381-98.

5. Fan M, Sun D, Zhou T, Heianza Y, Lv J, Li L, et al. Sleep patterns, genetic susceptibility, and incident cardiovascular disease: a prospective study of 385 292 UK biobank participants. *Eur Heart J*. (2020);41(11):1182-9.
